# Thyrotropin-releasing hormone analog as a stable upper airway-preferring respiratory stimulant with arousal properties

**DOI:** 10.1152/japplphysiol.00414.2022

**Published:** 2022-09-22

**Authors:** Wen-Ying Liu, Raina Ladha, Hattie Liu, Richard L. Horner

**Affiliations:** ^1^Department of Physiology, University of Toronto, Toronto, Ontario, Canada; ^2^Department of Medicine, University of Toronto, Toronto, Ontario, Canada

**Keywords:** genioglossus, obstructive sleep apnea, sleep, taltirelin, thyrotropin-releasing hormone

## Abstract

Taltirelin is a stable, brain-penetrating thyrotropin-releasing hormone (TRH) analog with minimal endocrine activity and potential respiratory stimulant properties. Taltirelin’s receptor target shows high differential expression at the hypoglossal motor nucleus, and local taltirelin microperfusion into the hypoglossal motor nucleus causes sustained tongue motor activation compared with the transient activating effects of TRH itself. Here, we performed a randomized, within-subject, repeated-measures design over six separate study days (separated by at least 72 h) in chronically instrumented male (*n* = 10) and female (*n* = 9) rats to identify effects on sleep and breathing. Vehicle controls or taltirelin (0.1 and 1 mg/kg) with and without trazodone (30 mg/kg) were administered by intraperitoneal injection. Trazodone was included due to clinical interest in the context of sleep apnea pharmacotherapy as it can suppress arousal without compromising pharyngeal muscle activity. Systemically administered taltirelin (1 but not 0.1 mg/kg) increased tonic and within-breath phasic tonic muscle activity compared with vehicle controls (*P* ≤ 0.007), with little or no changes in diaphragm amplitude or respiratory rate. Taltirelin also suppressed nonrapid eye movement (non-REM) sleep and increased wakefulness (*P* ≤ 0.037). Other indices of taltirelin-induced central nervous system arousal included increased trapezius muscle tone in non-REM sleep and decreased total electroencephalogram power and δ (0.5–4 Hz) power (*P* ≤ 0.046). These effects were especially apparent in non-REM sleep and not prevented by trazodone. These preclinical findings identify taltirelin as a stable upper airway-preferring respiratory stimulant with arousal properties, traits that have potential favorable relevance to some respiratory disorders but not others.

**NEW & NOTEWORTHY** One of the major goals for translational sleep science and medicine is to identify viable and tractable pharmacological targets for obstructive sleep apnea and other respiratory disorders of sleep or sedation. In the present preclinical study in rats, we performed a randomized, within-subject, repeated-measures design over six intervention study days in chronically instrumented male and female rats with systemic peripheral administration of vehicle controls, the thyrotropin-releasing hormone analog taltirelin at two doses, all with and without coadministered trazodone. Trazodone was included due to clinical interest in the context of sleep apnea pharmacotherapy as it can suppress arousal without compromising pharyngeal muscle activity. These preclinical findings newly identify taltirelin as a stable upper airway-preferring respiratory stimulant with arousal properties. These traits have potential favorable relevance to some respiratory disorders but not others, as identified and discussed.

## Graphical abstract

**Figure:**
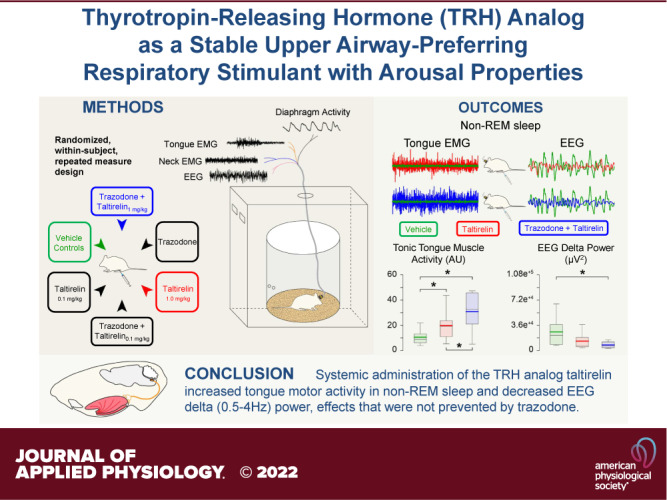

## INTRODUCTION

Obstructive sleep apnea (OSA) is a common and debilitating breathing disorder (1, 2) that remains undertreated due to poor compliance with the leading therapy, continuous positive airway pressure (3, 4). Identification of pharmacological agents to stimulate breathing and improve OSA is of high strategic, translational, and current interest (5, 6).

Findings from animal studies identified an endogenous noradrenergic excitatory drive that contributes to activation of the tongue musculature in wakefulness with this drive withdrawn in sleep (7–11), and a muscarinic receptor mechanism mediates strong tongue motor inhibition in rapid eye movement (REM) sleep (12). Those and other findings led to selection of cholinergic-noradrenergic drug combinations and successful OSA pharmacotherapy (13–17).

An unbiased screen recently identified additional pharmacological targets of high interest to modulate hypoglossal motor activity for potential OSA pharmacotherapy, and provided a database of these targets with associated Food and Drug Administration (FDA)-approved drugs (18). The aforementioned muscarinic and noradrenergic targets (M_2_ and α_1_ receptors respectively) that are strong controllers of hypoglossal motor activity in sleep, and successful targets in OSA pharmacotherapy, were identified from that screen, being expressed 3.6- and 3.7‐fold higher at the hypoglossal motor nucleus compared with the rest of the brain (18).

Notably, among other potential targets at the hypoglossal motor nucleus, thyrotropin-releasing hormone (TRH) receptor RNA also shows a high degree of differential expression at the hypoglossal motor nucleus compared with the rest of the brain (6.3‐fold), and higher than the 3.6- and 3.7-fold for M_2_ and α_1_ receptors at the hypoglossal motor nucleus (18). There are dense anatomical projections of TRH-positive neurons to the hypoglossal motor nucleus and identified excitatory effects on hypoglossal motoneurons (19–21). Overall, those findings stimulated an initial study using the TRH analog taltirelin to identify effects on pharyngeal muscle activity including the relatively selective activation of tongue motor activity in sleep with either local microperfusion into the hypoglossal motor nucleus or systemic (intraperitoneal) delivery in rats (22). Taltirelin is of further interest for the present study given it is a stable, brain-penetrating TRH-analog that is selective for nonendocrine actions (22–24).

In the present study, we performed a randomized, within-subject, repeated-measures design over six separate study days in chronically instrumented awake and sleeping rats (male and female) with combinations of systemically administered vehicle controls or taltirelin (two doses), with and without addition of trazodone. Trazodone was included due to clinical interest in the context of OSA pharmacotherapy because it can increase arousal threshold without compromising upper airway muscle activity (25–32); arousal threshold is one of the pathophysiological traits in OSA that is also amenable to pharmacotherapy (6, 33).

The findings of the present study identify the TRH analog taltirelin as a stable upper airway-preferring respiratory stimulant with arousal properties, traits that have potential favorable relevance to some respiratory disorders but not others.

## METHODS

### Ethical Approval

Procedures conformed to the recommendations of the Canadian Council on Animal Care and the University of Toronto Animal Care Committee approved the protocols. Experiments were performed on a total of 23 adult Wistar rats (14 males and 9 females, Charles River) but data from 4 males were excluded from the analysis due to dislodgment of the acrylic headpiece during connection that necessitated the termination of the experiments in those animals. As such the protocol (lasting at least 21 days per animal) was completed in 19 animals (82.6% success rate) comprising 10 males [mean body weight = 349.2 ± (SE) 7.3 g; range, 299–390 g] and 9 females (mean body weight = 256.3 ± 4.8 g; range, 225–288 g). Animals were housed under a 12/12-h light-dark cycle with lights on from 7:00 AM to 7:00 PM, and they had free access to food and water.

### Animal Preparation

General anesthesia was induced by inhaled isoflurane (3%–4%) with the animal in an induction chamber and anesthesia was then maintained via a mask placed over the snout (2.0%–3.0% isoflurane). Oxygen was administered to the inspired air (50% oxygen, balance air) throughout surgery. Buprenorphine (1 mg/kg, sc) and meloxicam (2 mg/kg, sc) were administered before surgery for analgesia, and meloxicam for the next 2 days after surgery. Effective general anesthesia was judged by abolition of the pedal withdrawal and corneal blink reflexes. During surgery, body temperature was maintained with a water pump and heating pad (T/Pump-Heat Therapy System, Gaymar, Orchard Park, NY). As previously described (22), the rats were then implanted with prepared electrodes for the chronic recording of the electroencephalogram (EEG) plus electromyographic (EMG) recordings from the tongue, diaphragm, and trapezius (neck) muscles.

At the end of surgery, the electrodes were connected to pins inserted into a miniature plug (STC-89PI-220ABS, Carleton University, Ottawa, ON, Canada). The plug was fixed to the skull with dental acrylic and anchor screws. After surgery, the rats were transferred to a clean cage and kept warm by a heating pad until full recovery as judged by normal motor activity, drinking, and eating. The rats were given soft mash food for the first day after surgery. The rats were then housed individually and recovered for a minimum of 7 days (range, 7–10 days) before the experiments were performed.

### Recordings and Protocol

For recordings, a lightweight shielded cable was connected to the plug on the rat’s head. The recording environment consisted of a large open-topped bowl (Rodent Bowl, MD-1514, BAS) housed within an electrically shielded and soundproofed cubicle (EPC-010, BRS/LVE), with the animals free from any disturbance and supplied with fresh bedding, food, and water. A video camera inside the cubicle allowed for continuous monitoring. For habituation, the rats were connected to the cable the day before the experiments, typically in the late afternoon from 4:00 PM to 5:00 PM with the experiments starting next day.

The experiments began at ∼9:00 AM and were performed during the day when the rats normally sleep. The electrophysiological signals were amplified and filtered (Super-Z head-stage amplifiers and BMA-400 amplifiers/filters, CWE Inc., Ardmore, PA). The EEG was filtered between 1 and 100 Hz, whereas the tongue, neck, and diaphragm EMGs were filtered between 100 and 1,000 Hz. The EMG signals were recorded at the same amplification across all experiments. The electrocardiogram was removed from the diaphragm signal using an electronic blanker (Model SB-1, CWE Inc.). The moving-time averages of the tongue and neck EMGs (time constant = 200 ms) and the diaphragm EMGs (time constant = 100 ms) were also obtained. The signals were digitized at a sampling rate of 2,000 Hz using a data acquisition system (CED 1401 and Spike 2 software, v.6, Cambridge Electronic Design Ltd., Cambridge, UK).

The protocol consisted of a randomized, within-subject, repeated-measures design over six separate intervention study days (separated by at least 72 h) in both the male and female rats with combinations of systemically administered intraperitoneal injections of vehicle controls or taltirelin (0.1 and 1.0 mg/kg), with and without addition of trazodone (30 mg/kg). Taltirelin (molecular weight: 456.46, Cat. No. 466799, US Biological Life Sciences) was dissolved in sterile 0.9% saline (Vehicle_1_) as described previously (22). Trazodone (molecular weight: 408.32, Cat. No. T6154, Sigma-Aldrich) was dissolved in dimethyl sulfoxide (DMSO, Vehicle_2_, sterile filtered, Cat. No. 3176, Tocris Bioscience) as in previous studies (34, 35). Applied doses were also based on previous studies (22, 24, 36–41). Each experimental day condition consisted of two intraperitoneal injections separated by 30 min: trazodone or vehicle control first at ∼9:00 AM followed by taltirelin (0.1 or 1.0 mg/kg) or vehicle control at ∼9:30 AM. The timings of drug administrations were selected based on pharmacokinetic data that peak plasma concentrations were occurring during the recording period (37, 40, 42). Data were collected over 5 h postinjection (∼10:00 AM to ∼3:00 PM). The primary analysis was performed on data collected from the 2nd to 4th hour postinjection, when responses (if any) would be expected to be observed (22, 24, 36–41). A secondary analysis was also performed on data collected in the 1st to 3rd and 3rd to 5th hours postinjection to test for potential maintenance or stability of responses over that period.

### Data Analysis

Sleep-wake states were identified visually from inspection of the neck EMG and the EEG and classified using standard criteria, with data analyses performed as previously described (12, 22). In summary, measurements of muscle activities within the identified sleep-wake states were made during all periods of wakefulness (>30 s in duration), non-REM sleep (> 60 s duration), and REM sleep (>30 s duration) (22). Data were included in the analyses of respiratory muscle activity only if they were obtained during unequivocal and clearly defined sleep-wake states; data obtained during transitional states, including drowsiness, arousals from sleep, and transitions from non-REM to REM sleep, were not included in the analyses.

The EMGs were analyzed from the respective moving average signal (above electrical zero) and were quantified in arbitrary units. Electrical zero was the voltage recorded with the amplifier inputs grounded. Tongue muscle EMG was quantified as mean tonic activity (i.e., minimal activity in expiration) and within-breath phasic activity (i.e., peak inspiratory activity – tonic activity). Mean neck muscle activity, mean diaphragm EMG amplitudes (i.e., phasic respiratory diaphragm activity), and respiratory rates were also calculated (12, 22). The EEG was also analyzed as previously described (12, 22). The EEG was sampled at 500 Hz and then analyzed via overlapping segments of 1,024 samples, windowed using a raised cosine (Hamming) function and subjected to a fast Fourier transform to yield the power spectrum (12, 22). The window was advanced in steps of 512 samples, and the mean power spectrum of the EEG signal for each 5-s epoch was calculated. The power contained within six frequency bands was recorded as absolute power and as a percentage of the total power of the signal. The band limits were δ_2_ (0.5–2 Hz), δ_1_ (2–4 Hz), θ (4–7.5 Hz), α (7.5–13.5 Hz), β_1_ (13.5–20 Hz), and β_2_ (20–30 Hz).

### Statistics

The analyses performed for each statistical test are included in the text where appropriate. For all comparisons, differences were considered significant if the null hypothesis was rejected at *P* < 0.05 using a two-tailed test. Where a two-way analysis of variance with one repeated measure (ANOVA-1RM) was performed, the factors were experimental condition (repeated measure) and sex (nonrepeated measure). The primary analyses were focused on effects in given sleep-wake states, principally non-REM sleep because it is the most stable physiological state to identify hypothesized effects (or not) on the output variables. Where a two-way analysis of variance with two repeated measures (ANOVA-2RM) was performed, the factors were data collected in the 1st to 3rd and 3rd to 5th hours postinjection (repeated measure) and experimental intervention such as vehicle plus taltirelin and taltirelin plus trazodone (repeated measure). Holm–Sidak post hoc comparisons were performed to determine significant differences between interventions or groups. The Pearson product-moment correlation coefficient (*r*) was also calculated between EEG δ power and the other parameters [i.e., tonic and within-breath phasic tongue motor activities, postural (neck) EMG activity, respiratory rate, and diaphragm amplitude] using both raw data and data normalized to the vehicle controls (i.e., Vehicle_1_ and Vehicle_2_) within each animal across the experimental conditions. Analyses were performed using Sigmaplot version 11 (Systat Software Inc., San Jose, CA).

## RESULTS

**Figure 1.**
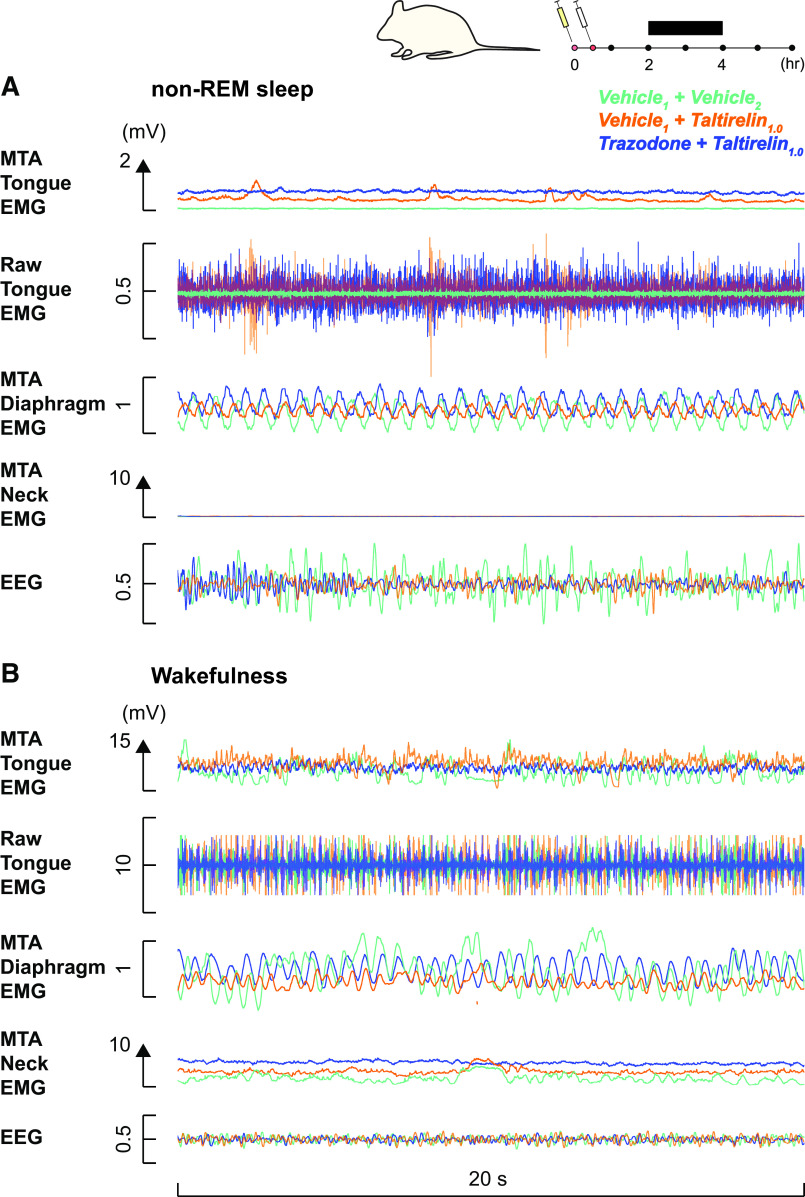
Example response to systemic administration of taltirelin across sleep-wake states across experimental conditions on separate days in one male subject. The traces are superimposed from the same rat in three of the different conditions for ease of comparison. Superimposition of the tongue electromyogram (EMG) traces with taltirelin (1 mg/kg) with and without trazodone, and vehicle controls, illustrates the increased tongue muscle activity in the presence of taltirelin that is most notable in non-rapid eye movement (non-REM) sleep (*A*) versus wakefulness (*B*). The electroencephalogram (EEG) traces are also superimposed to show the suppression of high-voltage slow-wave EEG activity in the presence of taltirelin during non-REM sleep. MTA, moving time average. See text for further details.

**Figure 2.**
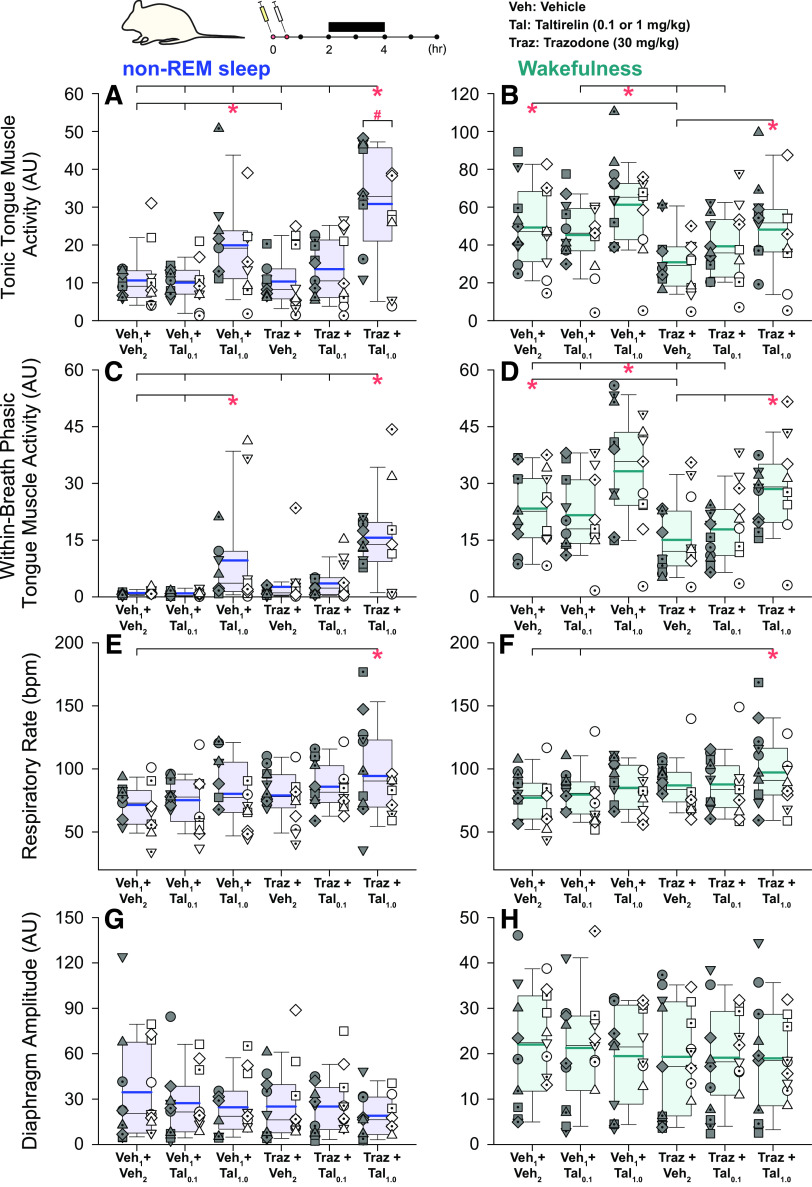
Individual and average effects of the different experimental interventions on tongue and diaphragm motor activities. Box (25th and 75th percentiles) and whisker (5th and 95th percentiles) plots show the individual and group data after intraperitoneal injections of taltirelin, with or without coadministration of trazodone, compared with the respective vehicle controls. Data are shown for non-rapid eye movement (non-REM) sleep (*left*) and wakefulness (*right*) for tonic tongue muscle activity (*A* and *B*), within-breath phasic tongue muscle activity (*C* and *D*), respiratory rate (*E* and *F*), and diaphragm amplitude (*G* and *H*). Individual data points from each animal are superimposed on these group data plots with each animal represented by a different symbol; the individual-specific symbols are consistent across the different experimental interventions on this and other figures. The box and whisker plots represent data from the group of 19 animals (both sexes, no individual symbols), with individual data in males (*n* = 10) and females (*n* = 9) on the *left* and *right*, respectively, of each group plot. The group mean and median are shown by the thicker and thinner lines. The symbols “*” and “#” indicate *P* < 0.05 between the indicated experimental conditions (*) or sex (#). AU, arbitrary units; bpm, breaths per minute; Tal, taltirelin; Traz, trazodone; Veh, vehicle. See text for further details.

**Figure 3.**
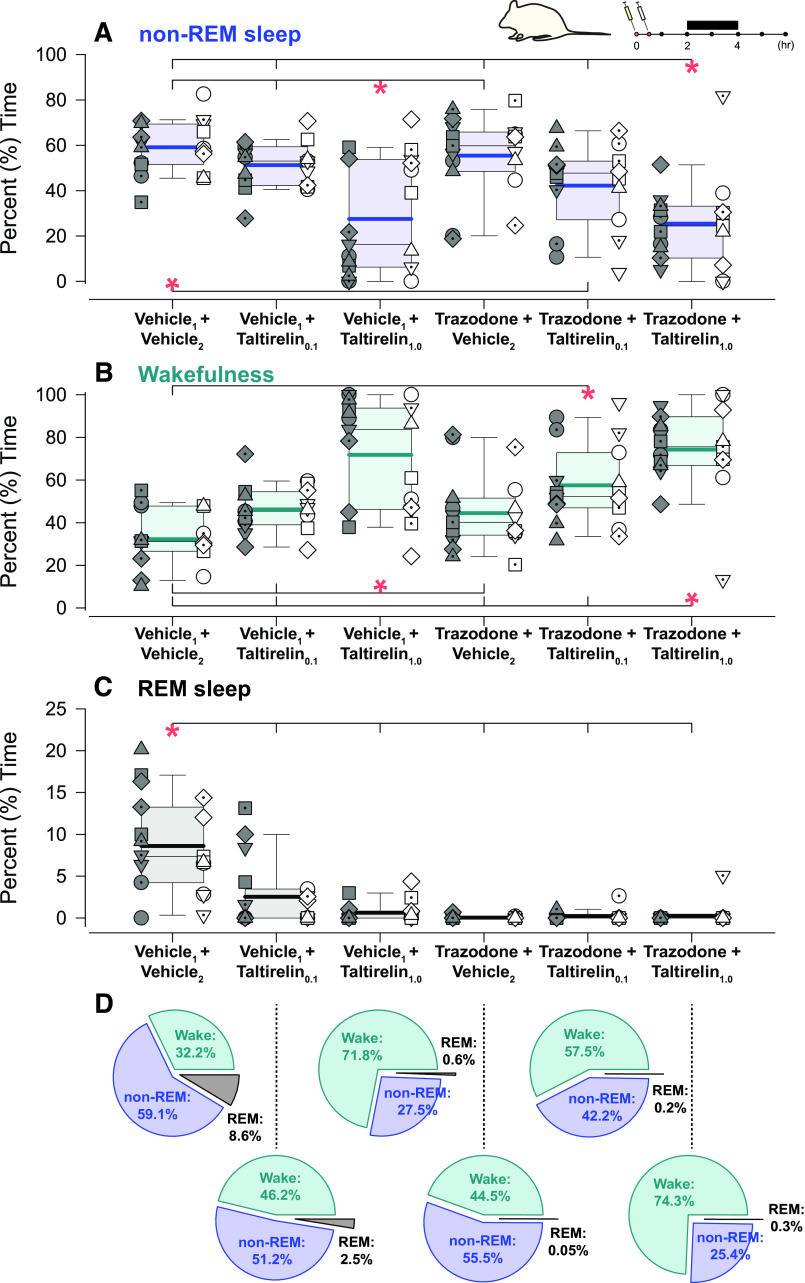
Individual and average effects of the different experimental interventions on the distribution of sleep-wake states. Box (25th and 75th percentiles) and whisker (5th and 95th percentiles) plots show the individual and group data after intraperitoneal injections of taltirelin, with or without coadministration of trazodone, compared with the respective vehicle controls. Data are shown for non-rapid eye movement (non-REM) sleep (*A*), wakefulness (*B*), and REM sleep (*C*), with relative proportions shown in pie charts (*D*). Individual data points from each animal are superimposed on these group data plots with each animal represented by a different symbol; the individual-specific symbols are consistent across the different experimental interventions on this and other figures. The box and whisker plots represent data from the group of 19 animals (both sexes, no individual symbols), with individual data in males (*n* = 10) and females (*n* = 9) on the *left* and *right*, respectively, of each group plot. The group mean and median are shown by the thicker and thinner lines. *P < 0.05 between the indicated experimental conditions. AU, arbitrary units; Tal, taltirelin; Traz, trazodone; Veh, vehicle. See text for further details.

**Figure 4.**
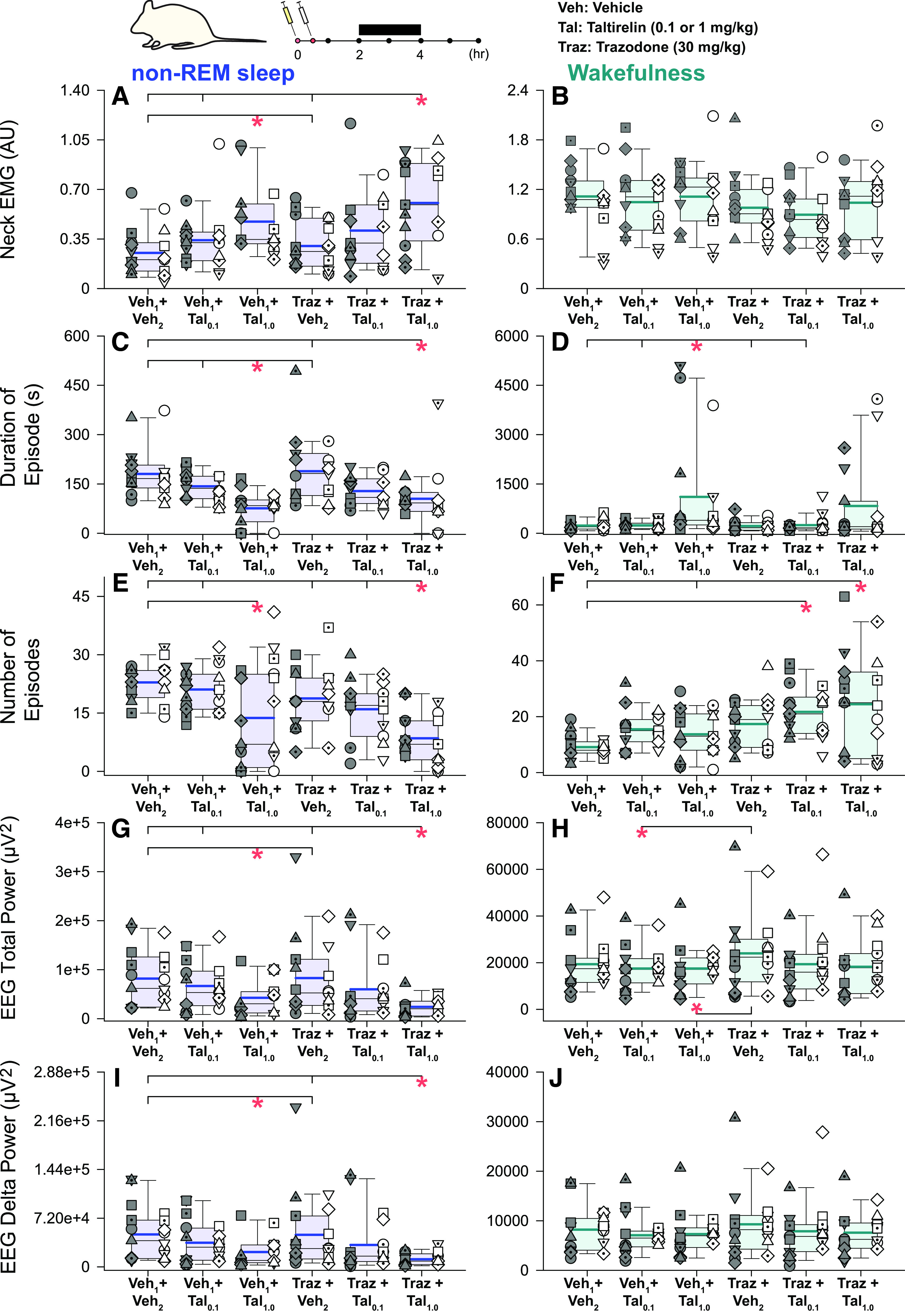
Individual and average effects of the different experimental interventions on other indices of central nervous system arousal. Box (25th and 75th percentiles) and whisker (5th and 95th percentiles) plots show the individual and group data after intraperitoneal injections of taltirelin, with or without coadministration of trazodone, compared with the respective vehicle controls. Data are shown for non-rapid eye movement (non-REM) sleep (*left*) and wakefulness (*right*) for neck electromyogram (EMG) activity (*A* and *B*), duration and number of sleep-wake episodes (*C*–*F*), total electroencephalogram (EEG) power (*G* and *H*), and EEG δ (0.5–4 Hz) power (*I* and *J*). Individual data points from each animal are superimposed on these group data plots with each animal represented by a different symbol; the individual-specific symbols are consistent across the different experimental interventions on this and other figures. The box and whisker plots represent data from the group of 19 animals (both sexes, no individual symbols), with individual data in males (*n* = 10) and females (*n* = 9) on the *left* and *right*, respectively, of each group plot. The group mean and median are shown by the thicker and thinner lines. *P < 0.05 between the indicated experimental conditions. AU, arbitrary units; Tal, taltirelin; Traz, trazodone; Veh, vehicle. See text for further details.

Figure 1 shows an example of increased mean tonic tongue motor activity following systemic administration of taltirelin via intraperitoneal injection, with or without co-administration of trazodone, compared with the vehicle controls. Note the increased tongue muscle activation with taltirelin is most notable in non-REM sleep. Also apparent in Fig. 1 is the suppression of high-voltage slow-wave EEG activity in the presence of taltirelin during non-REM sleep. The averaged individual and group data across sleep-wake states in each rat are shown in Figs. 2, 3, and 4. There was a marked suppression of REM sleep in the presence of taltirelin (Fig. 3) such that motor activities were only analyzed and reported for periods of non-REM sleep and wakefulness.

### Tonic Tongue Muscle Activity

Figure 2, and the statistical analysis, identified a significant effect of experimental intervention on the magnitude of tonic tongue muscle activity in non-REM sleep (*F*_5,79 _= 18.06, *P* < 0.001, two-way ANOVA-1RM). Post hoc analyses identified that tonic tongue muscle activity in the combined presence of trazodone and taltirelin (1 mg/kg) was greater than all the other conditions including the same dose taltirelin without trazodone (each *t* ≥ 3.56, all *P* ≤ 0.006, see symbol “*,” Fig. 2*A*). Taltirelin alone also increased tonic tongue motor activity in non-REM sleep compared with the respective vehicle controls with and without 0.1 mg/kg taltirelin or trazodone (each *t* ≥ 3.536, all *P* ≤ 0.005, also see symbol “*,” Fig. 2*A*).

There was also a significant effect of experimental intervention on the magnitude of tonic tongue muscle activity in wakefulness, notably with increased activity with taltirelin (1 mg/kg) in the presence or absence of trazodone compared with either the lesser dose of taltirelin (0.1 mg/kg) or the respective vehicle controls (post hoc tests: *t* ≥ 3.13, *P* ≤ 0.026 after two-way ANOVA-1RM: *P* < 0.001, see symbols “*,” Fig. 2*B*). There was also a suppression of tonic tongue motor activity in wakefulness observed with trazodone alone compared with the vehicle control (*t* = 3.62, *P* = 0.007, see symbol “*” in Fig. 2*B*).

An effect of sex on the response to the experimental condition was also identified in non-REM sleep (*F*_5,79 _= 2.91, *P* = 0.018, two-way ANOVA-1RM) but not wakefulness (*P* = 0.658). Post hoc analysis identified that such a sex effect in non-REM sleep only occurred in the combined presence of trazodone and taltirelin (1 mg/kg) with activity being larger in males than females (*t* = 2.87, *P* = 0.006, see symbol “#,” Fig. 2*A*).

### Within-Breath Phasic Muscle Activity

There was also a significant effect of experimental intervention on the magnitude of within-breath phasic tongue muscle activity in non-REM sleep (*F*_5,79 _= 12.58, *P* < 0.001, two-way ANOVA-1RM) that did not depend on animal sex (*F*_5,79 _= 0.16, *P* = 0.977), i.e., there was no statistically significant interaction.

Post hoc analyses identified that within-breath phasic tongue muscle activity in the combined presence of trazodone and taltirelin (1 mg/kg) in non-REM sleep was greater than the other conditions (each *t* ≥ 5.26, all *P* ≤ 0.001, see symbol “*,” Fig. 2*C*) except 1 mg/kg taltirelin alone (*t* = 2.57, *P* = 0.092). Taltirelin alone also increased within-breath phasic tongue muscle activity compared with the respective vehicle controls and 0.1 mg/kg taltirelin in non-REM sleep (each *t* ≥ 3.546, all *P* = 0.007, see symbol “*,” Fig. 2*C*). Statistically significant changes identified in wakefulness are also shown in Fig. 2*D*.

### Respiratory Rate

There was a statistically significant main effect of sex on respiratory rate in non-REM sleep (*F*_1,17 _= 7.35, *P* = 0.015, 2-way ANOVA-1RM) but not in wakefulness (*F*_1,17 _= 3.58, *P* = 0.076, two-way ANOVA-1RM), likely due a masking effect of the waking state and behaviors. In non-REM sleep, post hoc analysis identified increased respiratory rates in males versus females (*t* = 2.69, *P* = 0.015). Respiratory rate was relatively stable across the various interventions although there was a significant increase in respiratory rate detected in the combined presence of taltirelin (1 mg/kg) and trazodone in non-REM sleep and wakefulness compared with the vehicle controls (post hoc tests: *t* ≥ 3.49, *P* ≤ 0.012 after two-way ANOVA-1RM: *P* ≤ 0.001, see symbol “*,” Fig. 2, *E* and *F*).

There was no effect of experimental intervention that depended on sex, i.e., there was no statistically significant interaction in waking or non-REM sleep (*F*_5,79_ ≤ 1.64, *P* ≥ 0.160, two-way ANOVA-1RM).

### Diaphragm Amplitude

There was no effect of sex (*P* ≥ 0.272, two-way ANOVA-1RM), experimental intervention (*P* ≥ 0.063), or interaction of sex by intervention (*P* ≥ 0.520) on the magnitude of diaphragm activity in non-REM sleep or wakefulness (Fig. 2, *G* and *H*).

### Sleep-Wake States

There was a statistically significant effect of experimental intervention on the percent distribution of wakefulness, non-REM sleep, and REM sleep (each *P* < 0.001, two-way ANOVA-1RM) but there was no main effect of sex (each *P* ≥ 0.157) nor an interaction of sex on the responses to the interventions (each *P* ≥ 0.073). Overall, taltirelin exhibited properties of a central nervous system stimulant with significant suppression of non-REM sleep and increased wakefulness, an effect that was not mitigated by trazodone (see symbols “*” in Fig. 3, *A* and *B*, each *P* ≤ 0.037). In addition to the suppression of non-REM sleep, there was minimal REM sleep or complete REM sleep suppression in the presence of taltirelin, again an effect that was not mitigated by trazodone and even occurred with trazodone alone plus vehicle (see symbols “*” in Fig. 3*C*, each *P* ≤ 0.001).

### Other Indices of Central Nervous System Arousal

There was a significant effect of experimental intervention on neck EMG that was detected in non-REM sleep (*F*_5,79 _= 7.89, *P* < 0.001, two-way ANOVA-1RM) but not wakefulness (*F*_5,85 _= 1.73, *P* = 0.137), but there was no main effect of sex, and no interaction of sex and intervention across states (each *P* ≥ 0.071). Post hoc analyses identified a stimulating effect of taltirelin on neck motor activity in non-REM sleep, and still occurred when trazodone was administered with taltirelin (see symbols “*” in Fig. 4, *A* and *B*, each *P* ≤ 0.027).

In addition to the changes in percent sleep-wake states identified in Fig. 3, there were changes in both the duration and number of non-REM sleep and wake episodes in response to the experimental interventions (all ≤ 0.004, two-way ANOVA-1RM). Overall non-REM sleep episodes were shorter and less in number in the presence of taltirelin, with corresponding increases in the number and duration of waking episodes (each *P* ≤ 0.044, see symbols “*” in Fig. 4, *C*–*F*).

Total EEG power in non-REM sleep and wakefulness was also affected by the experimental interventions (both *P* ≤ 0.018, two-way ANOVA-1RM), but there was no main effect of sex (each *P* ≥ 0.356) nor an interaction of sex on the responses to the interventions (each *P* ≥ 0.607). Post hoc analyses identified an EEG activating effect, as reflected by lower total EEG power, of taltirelin, which also occurred during coadministration of trazodone; this effect was most prominently observed in non-REM sleep but also detected in wakefulness (see symbols “*” in Fig. 4, *G* and *H*, each *P* ≤ 0.030). Further analysis also identified that EEG δ power was significantly attenuated in the presence of taltirelin in non-REM sleep (see symbols “*” in Fig. 4*I*, each *P* ≤ 0.046 after two-way ANOVA-1RM: *P* < 0.001). There was no effect of experimental intervention on EEG delta power in wakefulness (*P* = 0.161, two-way ANOVA-1RM).

**Figure 5.**
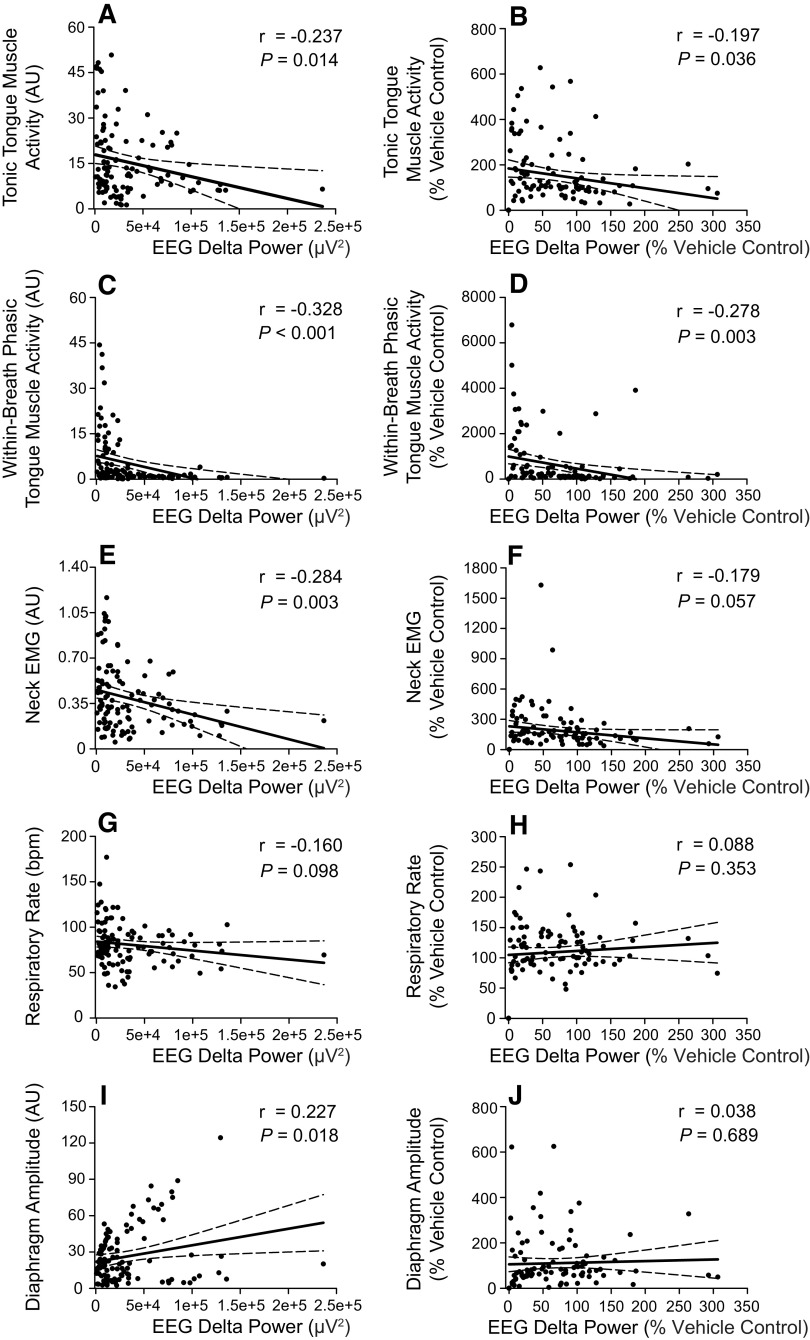
Correlations between EEG delta power and motor activities in non-rapid eye movement (non-REM) sleep. The plots and statistical analyses show the relationships between electroencephalogram (EEG) δ power and tonic (*A* and *B*) and within-breath phasic (*C* and *D*) tongue motor activity, postural (neck) electromyogram (EMG) activity (*E* and *F*), respiratory rate (*G* and *H*), and diaphragm amplitude (*I* and *J*). The individual data points show raw values (*left*) and values normalized to the vehicle controls (i.e., Vehicle_1_ and Vehicle_2_) within each animal across the experimental conditions (*right*).

To assess the relationship between sleep “depth” as reflected by EEG δ power in non-REM sleep and the magnitude of the other parameters, we performed correlations as shown in Fig. 5. These data show statistically significant inverse relationships between EEG δ activity and both tonic and within-breath phasic tongue motor activity for both raw data and the data normalized to the vehicle controls (Fig. 5, *A*–*D*, range of *r* = −0.328 to −0.197, range of *P* = 0.036 to <0.001), i.e., less δ activity was associated with higher tongue motor activity. Some significant relationships were also apparent for neck EMG activity and diaphragm amplitude (Fig. 5, *E* and *I*, respectively).

**Figure 6.**
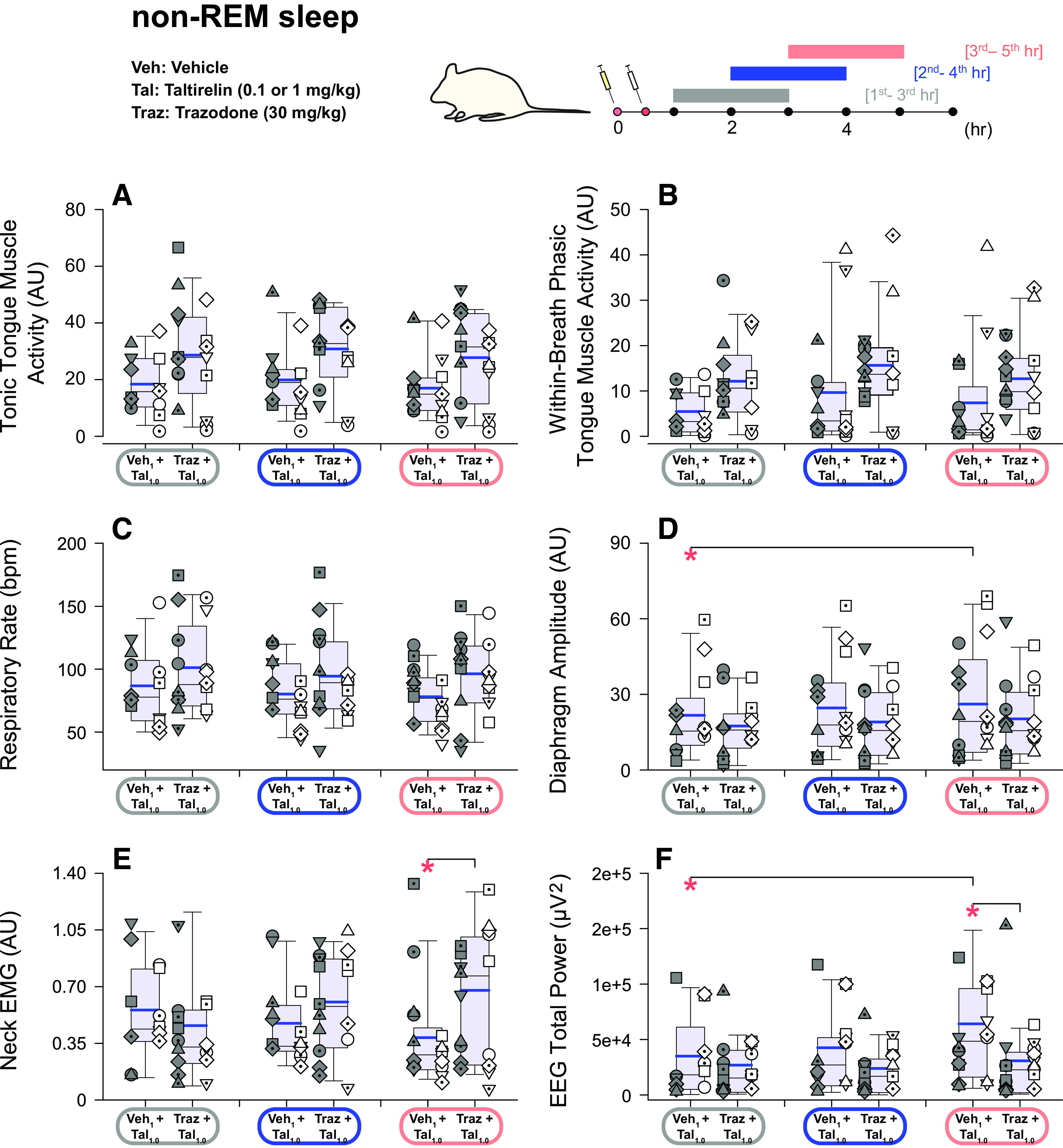
Effects of taltirelin with and without trazodone on tongue, diaphragm, and postural (neck) motor activity and electroencephalogram power. Box (25th and 75th percentiles) and whisker (5th and 95th percentiles) plots show group data (*n* = 19, 10 males and 9 females, no individual symbols) from non-rapid eye movement (non-REM) sleep after intraperitoneal injections of taltirelin with or without coadministration of trazodone. Individual data in males (*n* = 10) and females (*n* = 9) are shown on the *left* and *right*, respectively, of each group plot. Data are shown for the 1st to 3rd and 3rd to 5th hours postinjection, in comparison to the 2nd to 4th hours used in the major analyses. The mean and median are shown by the thicker and thinner lines. *P < 0.05 between the indicated conditions. AU, arbitrary units; EEG, electroencephalogram; EMG, electromyogram; Tal, taltirelin; Traz, trazodone; Veh, vehicle. See text for further details.

The primary analysis was performed on data collected from the 2nd to 4th hour postinjection (Figs. 2, 3, and 4), when responses (if any) would be expected to be observed. A secondary analysis was also performed on data collected in the 1st to 3rd and 3rd to 5th hours postinjection to test for potential maintenance or stability of responses over that period. These data are shown in Fig. 6. For tonic and within-breath phasic tongue motor activity, and respiratory rate, these data showed main effects that were not different between the earlier and later periods of analysis (*P* = 0.585, 0.206 and 0.590 respectively, two-way ANOVA-2RMs) and no interaction with the different interventions (*P* = 0.929, 0.831, and 0.277 respectively). There were no differences in neck EMG activity in the 1st to 3rd and 3rd to 5th hours of analysis (*P* = 0.742) but there were differences identified in these periods for specific interventions for diaphragm amplitude, neck EMG and EEG power (each *P* ≤ 0.05, see symbols “*” in Fig. 6).

## DISCUSSION

Here we show from a randomized, within-subject, repeated-measures design that systemic administration of 1 mg/kg taltirelin (but not 0.1 mg/kg) increased tonic and within-breath phasic tongue muscle activity in male and female rats with little or no changes in diaphragm amplitude or respiratory rate; sex differences were generally absent. Taltirelin also suppressed non-REM and REM sleep and increased wakefulness, effects suggestive of brain arousal. The central nervous system stimulant properties of taltirelin were also supported by increased postural muscle tone, as identified from the trapezius muscle in non-REM sleep, and the decreased total EEG power and δ (0.5–4 Hz) power with taltirelin. These stimulant effects were especially apparent in non-REM sleep and were not prevented by trazodone. Trazodone was included as an intervention in this study given the central nervous system stimulant properties of taltirelin (22, 23, 43, 44). Moreover, there is clinical interest in trazodone in the armamentarium for OSA pharmacotherapy because trazodone can increase arousal threshold without compromising upper airway muscle activity (25–32); arousal threshold constitutes a predisposing factor for OSA pathophysiology and a pharmacologically modifiable trait (6, 33) with trazodone being one of the most prescribed sleep-promoting drugs (45, 46). Overall, the characterization of taltirelin as an upper airway-preferring respiratory stimulant with arousal properties suggests potential utility in some clinical situations that remain to be determined. These may include postoperative recovery and upper airway management, and/or to counter opioid or sedative-induced respiratory and arousal-state depression in the managed clinical setting (43, 47–49). The results of this preclinical study suggest, however, that taltirelin may not be useful by itself for OSA pharmacotherapy.

A notable feature of the present study, in addition to the pharyngeal muscle activating effects of taltirelin, was that the alerting effects of taltirelin were not mitigated by trazodone (Figs. 1, 3, and 4). Trazodone also contributed to the suppression of REM sleep. Trazodone is a tricyclic antidepressant, and although REM sleep suppression is a common consequence of their administration, trazodone has reportedly little effect on REM sleep in humans, or small decreases, with increased slow wave activity also commonly observed (46). The dose of trazodone was based on previous studies in rats (38–41). Two studies using smaller doses of trazodone in rats (10 mg/kg and 20 mg/kg) did show enhanced non-REM sleep with no effects on REM sleep (50, 51). Titrating doses of trazodone to find an optimal sleep-promoting dose may have improved sleep with taltirelin in the present study but does not change the interpretation that taltirelin is an upper airway preferring respiratory stimulant with arousal properties. Nor does it change the interpretation that trazodone does not compromise upper airway muscle activity. Other sedative hypnotics such as those targeting γ-aminobutyric acid (GABA)_A_ receptors can increase hypoglossal and tongue motor activity in rats and promote sedation (52–54) but are not well studied, nor currently favored, in the context of OSA pharmacotherapy (6, 25, 26).

We also assessed the relationship between sleep “depth” as reflected by EEG δ power in non-REM sleep and the magnitude of the tongue, diaphragm, and neck motor activities across the interventions. These data showed statistically significant inverse relationships between delta activity and both tonic and within-breath phasic tongue motor activities, i.e., less δ activity was associated with higher tongue motor activity. This finding is interesting but should be interpreted with caution. For example, it cannot be determined if the coincident tongue motor activation and central nervous system stimulation are independent effects of taltirelin or if one effect influences the other, i.e., if lightening of sleep is an actual cause of tongue motor activation or at least a contributing factor. The potential dissociation of these two effects using a hypnotic that selectively boosts slow wave activity would be a fruitful area of investigation to address this question. Nevertheless, the findings that taltirelin at the hypoglossal motor nucleus elicits sustained tongue motor activating effects (22) and that TRH receptor RNA shows a high degree of differential expression at the hypoglossal motor nucleus (6.3-fold) compared with the rest of the brain (18) can be used to reasonably explain at least a component of the tongue motor response to systemically administered taltirelin. It is also noted that the correlation coefficients for the relationships between δ power and tongue motor activity are relatively low (range of *r* from −0.197 to −0.328, Fig. 5, *A*–*D*), suggesting that a correspondingly low proportion of the variance in tongue motor activity is statistically related to variations in δ power. Overall, these correlative data cannot exclude a contribution of lightened sleep to tongue motor activation, although any cause-and-effect relationship, or its relative importance to the overall magnitude of response, cannot be directly determined.

In non-REM sleep, the combined presence of taltirelin (1 mg/kg) and trazadone led to increased tonic tongue motor activity compared with 1 mg/kg taltirelin alone, but this indicator of a potentiated effect did not occur for within-breath phasic tongue muscle activity in non-REM sleep or either measure of tongue motor activity in wakefulness (Fig. 2). This increased tonic motor activity with 1 mg/kg taltirelin plus trazadone compared with 1 mg/kg taltirelin alone also did not occur for neck EMG, respiratory rate, or diaphragm amplitude in non-REM or wakefulness (Figs. 2 and 4); i.e., was specific to tonic tongue motor activity in non-REM sleep. It is possible that the greater tonic tongue motor response with trazadone was due to its serotonergic agonist properties and is consistent with the improvements in sleep-disordered breathing in bulldogs (29) and some improvements in OSA severity in human studies with serotonergic agents, although they appear to be less effective than cholinergic-noradrenergic drug combinations (6).

Deep non-REM sleep is the most stable physiological state to identify effects on breathing and autonomic control, with wakefulness potentially leading to masking effects (55). It is in this context that, in our opinion, the stronger effects of taltirelin in non-REM sleep are best interpreted. The effects of taltirelin as an upper airway preferring respiratory stimulant with arousal properties were most visibly obvious and statistically significant in non-REM sleep, but signs of such effects were also apparent visually and statistically in wakefulness (Figs. 2 and 4). We do not interpret these data to suggest that taltirelin is exerting such respiratory and arousal effects preferentially in non-REM sleep, or is gated to states of reduced brain arousal, but that wakefulness can simply partially mask these effects due to the inherent variability associated with the waking state and behaviors. It is with this understanding that such mixed properties of taltirelin as an upper airway preferring respiratory stimulant with arousal properties may be harnessed to potential beneficial effect in select clinical scenarios, with the potential to depress the arousal properties with specifically chosen pharmacological strategies, receptor targets, and doses as appropriate or needed.

The primary analysis was performed on data collected from the 2nd to 4th hour postinjection (Figs. 2–4), when responses (if any) would be expected to be observed (22, 24, 36–41). A secondary analysis was also performed on data collected in the 1st to 3rd and 3rd to 5th hours postinjection to test for potential maintenance or stability of responses over that period (Fig. 6). Some of the differences identified in Fig. 6 between the 1st to 3rd and 3rd to 5th hours occurred for neck motor activity, EEG power, and diaphragm amplitude but not the primary variables—tonic and within-breath phasic tongue motor activity, and respiratory rate—which were similar. Overall, these data and additional analysis showed similar tongue motor activation effects in the earlier and later periods of analysis, and as such stability of responses over the period.

The motor activating effects of taltirelin on components of the respiratory network were not confined to the tongue musculature but increases in respiratory rate were sometimes observed. In the context of such effects on respiratory rate, neurons of the nucleus tractus solitarii are immunoreactive for TRH, and TRH increases their rhythmic activity (56). Activation of respiratory neurons in the nucleus tractus solitarii in mice has also been shown to increase respiratory rate (57) and this effect may underlie the increased respiratory rate also observed after intracerebroventricular injections of TRH in rats (58).

Taltirelin is stated to exhibit strong selectivity for non-endocrine (neural) actions but minimal endocrine activity (23, 24, 59). In terms of specific relative numerical differences, taltirelin is also stated to be 10–100 times more potent as a central nervous system stimulant with 5–10 times lower endocrine action (23, 24, 60). This strong relative imbalance is thought to be due to taltirelin having low affinity but high stability for TRH receptors in the central nervous system leading to potent and long-lasting neural effects, in contrast to low affinity for TRH receptors in the pituitary and thus comparably weaker endocrine effects (23, 24, 59). Persistence of activity through resistance to metabolic degradation may be the key feature of the central nervous system stimulant properties of analogues such as taltirelin. Taltirelin is more metabolically stable than native TRH such that over time more of the stable analog in the circulation is able to penetrate the blood-brain barrier and thus exert prolonged neural effects (61).

Levels of thyroid activity and function through measures of thyroid-stimulating hormone (TSH), triiodothyronine (T3), and thyroxine (T4) were not performed in this study as this would have required additional instrumentation and procedures in these chronic experiments lasting several weeks. Oral administration of taltirelin has been shown to increase TSH, T3, and T4 (as expected) but such changes were within the normal ranges when applied at 5 mg once a day or 2.5 mg twice a day for 2 wk, with normal cardiovascular, body temperature, and blood and urine analyses (24). In the present study, taltirelin was administered at lower doses (1.0 or 0.1 mg/kg) and less often (once per day on several occasions but each separated by at least 3 days). As such our interventions may be expected to also elicit changes in TSH, T3, and T4 (if any) but if so, also likely within the normal range, i.e., as in the aforementioned study with higher daily doses.

It is also unknown if taltirelin altered body temperature and/or metabolism in the present study. Systemically administered TRH and taltirelin can increase body temperature as well as locomotor activity (24, 36, 60, 62), effects that are also likely mediated through its central nervous stimulant properties. Such potential effects on body temperature and/or activity may have contributed to effects on breathing (56, 57) and increased arousal (60, 63, 64). A comprehensive study in hamsters identified that intraperitoneal injections of 5 mg/kg TRH did not change body temperature, although increases of (<1°C) were observed with 50 mg/kg but only in the first hour postinjection and not thereafter (62). In that study neither 5 mg/kg nor 50 mg/kg TRH influenced motor activity after the first hour postinjection, and 5 mg/kg TRH increased oxygen consumption in the first hour postinjection but not thereafter (62). It is possible that taltirelin may affect body temperature, activity, and/or metabolism but further comprehensive studies in rats are needed, and at the taltirelin dose (1 mg/kg) used in the present study. In a rat model of motor ataxia, neither intraperitoneal injection of TRH (10 mg/kg) nor taltirelin (1 mg/kg) altered body temperature, arterial Pco_2_, Po_2_, or pH (65). In the present study, the only change in respiratory rate and/or diaphragm amplitude observed across all the protocols was increased respiratory rate in the combined presence of trazodone and 1 mg/kg taltirelin; effects on tongue motor activity were observed more consistently (Fig. 2) supporting the notion that at this dose taltirelin is more of an upper airway-preferring respiratory stimulant. Overall, the broader effects of taltirelin on sleep, breathing, behavior, and integrative physiology such as metabolism across the 24-h period with acute or chronic daily administration need to be determined.

In summary, it remains to be determined if this preclinical characterization of taltirelin as a stable upper airway-preferring respiratory stimulant with arousal properties has potential translational significance to clinical scenarios. Such scenarios may include postoperative recovery and upper airway management, and/or to counter opioid or sedative-induced respiratory and arousal-state depression in the managed clinical setting. In such cases the identified combination of properties may prove beneficial (43, 47–49). As it stands, however, the mixed upper airway-preferring respiratory stimulant and arousal properties may not be suitable for OSA pharmacology unless the arousal properties can be selectively diminished with a safe strategy that spares any respiratory depression.

## GRANTS

This work was supported by funds from Canadian Institutes of Health Research Grants PJT-153243 and PJT-180288 (to R.L.H.) and the National Sanitarium Association Innovative Research Program Grant 497478 (to R.L.H.). R.L.H. was supported by Tier I Canada Research Chair in Sleep and Respiratory Neurobiology Fund 950-229813.

## DISCLOSURES

No conflicts of interest, financial or otherwise, are declared by the authors.

## AUTHOR CONTRIBUTIONS

W.-Y.L., R.L., H.L., and R.L.H. conceived and designed research; W.-Y.L., R.L., and H.L. performed experiments; W.-Y.L., R.L., H.L., and R.L.H. analyzed data; W.-Y.L., H.L., and R.L.H. interpreted results of experiments; W.-Y.L., H.L., and R.L.H. prepared figures; W.-Y.L., H.L., and R.L.H. drafted manuscript; W.-Y.L., R.L., H.L., and R.L.H. edited and revised manuscript; W.-Y.L., R.L., H.L., and R.L.H. approved final version of manuscript.

## References

[1] Benjafield AV, Ayas NT, Eastwood PR, Heinzer R, Ip MSM, Morrell MJ, Nunez CM, Patel SR, Penzel T, Pepin JL, Peppard PE, Sinha S, Tufik S, Valentine K, Malhotra A. Estimation of the global prevalence and burden of obstructive sleep apnoea: a literature-based analysis. Lancet Respir Med 7: 687–698, 2019. doi:10.1016/S2213-2600(19)30198-5. 31300334 PMC7007763

[2] Peppard PE, Young T, Barnet JH, Palta M, Hagen EW, Hla KM. Increased prevalence of sleep-disordered breathing in adults. Am J Epidemiol 177: 1006–1014, 2013. doi:10.1093/aje/kws342. 23589584 PMC3639722

[3] Weaver TE, Grunstein RR. Adherence to continuous positive airway pressure therapy: the challenge to effective treatment. Proc Am Thorac Soc 5: 173–178, 2008. doi:10.1513/pats.200708-119MG. 18250209 PMC2645251

[4] Sawyer AM, Gooneratne NS, Marcus CL, Ofer D, Richards KC, Weaver TE. A systematic review of CPAP adherence across age groups: clinical and empiric insights for developing CPAP adherence interventions. Sleep Med Rev 15: 343–356, 2011. doi:10.1016/j.smrv.2011.01.003. 21652236 PMC3202028

[5] Kim LJ, Freire C, Fleury Curado T, Jun JC, Polotsky VY. The role of animal models in developing pharmacotherapy for obstructive sleep apnea. J Clin Med 8: 2049, 2019. doi:10.3390/jcm8122049. PMC6947279 31766589

[6] Taranto-Montemurro L, Messineo L, Wellman A. Targeting endotypic traits with medications for the pharmacological treatment of obstructive sleep apnea. A review of the current literature. J Clin Med 8: 1846, 2019. doi:10.3390/jcm8111846. 31684047 PMC6912255

[7] Fenik VB, Davies RO, Kubin L. Noradrenergic, serotonergic and GABAergic antagonists injected together into the XII nucleus abolish the REM sleep-like depression of hypoglossal motoneuronal activity. J Sleep Res 14: 419–429, 2005. doi:10.1111/j.1365-2869.2005.00461.x. 16364143

[8] Chan E, Steenland HW, Liu H, Horner RL. Endogenous excitatory drive modulating respiratory muscle activity across sleep-wake states. Am J Respir Crit Care Med 174: 1264–1273, 2006. doi:10.1164/rccm.200605-597OC. 16931636

[9] Fenik VB, Davies RO, Kubin L. REM sleep-like atonia of hypoglossal (XII) motoneurons is caused by loss of noradrenergic and serotonergic inputs. Am J Respir Crit Care Med 172: 1322–1330, 2005. doi:10.1164/rccm.200412-1750OC. 16100007 PMC5222563

[10] Song G, Poon CS. α_2_-Adrenergic blockade rescues hypoglossal motor defense against obstructive sleep apnea. JCI Insight 2: e91456, 2017. doi:10.1172/jci.insight.91456. 28239660 PMC5313063

[11] Fenik VB, Rukhadze I. Activity of pontine A7 noradrenergic neurons is suppressed during REM sleep. J Appl Physiol (1985) 133: 130–143, 2022. doi:10.1152/japplphysiol.00771.2021. 35616303 PMC9255708

[12] Grace KP, Hughes SW, Horner RL. Identification of the mechanism mediating genioglossus muscle suppression in REM sleep. Am J Respir Crit Care Med 187: 311–319, 2013. doi:10.1164/rccm.201209-1654OC. 23220910

[13] Taranto-Montemurro L, Messineo L, Sands SA, Azarbarzin A, Marques M, Edwards BA, Eckert DJ, White DP, Wellman A. The combination of atomoxetine and oxybutynin greatly reduces obstructive sleep apnea severity: a randomized, placebo-controlled, double-blind crossover trial. Am J Respir Crit Care Med 199: 1267–1276, 2019. doi:10.1164/rccm.201808-1493OC. 30395486 PMC6519859

[14] Taranto-Montemurro L, Messineo L, Azarbarzin A, Vena D, Hess LB, Calianese NA, White DP, Wellman A, Sands SA. Effects of the combination of atomoxetine and oxybutynin on osa endotypic traits. Chest 157: 1626–1636, 2020. doi:10.1016/j.chest.2020.01.012. 32006590 PMC7268440

[15] Lim R, Messineo L, Grunstein RR, Carberry JC, Eckert DJ. The noradrenergic agent reboxetine plus antimuscarinic hyoscine butylbromide reduces sleep apnoea severity: a double-blind, placebo-controlled, randomised crossover trial. J Physiol 599: 4183–4195, 2021. doi:10.1113/JP281912. 34174090

[16] Aishah A, Lim R, Sands SA, Taranto-Montemurro L, Wellman A, Carberry JC, Eckert DJ. Different antimuscarinics when combined with atomoxetine have differential effects on obstructive sleep apnea severity. J Appl Physiol (1985) 130: 1373–1382, 2021. doi:10.1152/japplphysiol.01074.2020. 33734828 PMC8424567

[17] Perger E, Taranto Montemurro L, Rosa D, Vicini S, Marconi M, Zanotti L, Meriggi P, Azarbarzin A, Sands SA, Wellman A, Lombardi C, Parati G. Reboxetine plus oxybutynin for obstructed sleep apnea treatment a 1-week randomized, placebo-controlled, double-blind crossover trial. Chest 161: 237–247, 2022. doi:10.1016/j.chest.2021.08.080.34543665

[18] Horner RL, Grace KP, Wellman A. A resource of potential drug targets and strategic decision-making for obstructive sleep apnoea pharmacotherapy. Respirology 22: 861–873, 2017. doi:10.1111/resp.13079. 28544082 PMC5515492

[19] Bayliss DA, Viana F, Kanter RK, Szymeczek-Seay CL, Berger AJ, Millhorn DE. Early postnatal development of thyrotropin-releasing hormone (TRH) expression, TRH receptor binding, and TRH responses in neurons of rat brainstem. J Neurosci 14: 821–833, 1994. doi:10.1523/JNEUROSCI.14-02-00821.1994. 8301363 PMC6576815

[20] Rekling JC, Funk GD, Bayliss DA, Dong XW, Feldman JL. Synaptic control of motoneuronal excitability. Physiol Rev 80: 767–852, 2000. doi:10.1152/physrev.2000.80.2.767. 10747207 PMC4764886

[21] Johansson O, Hokfelt T, Pernow B, Jeffcoate SL, White N, Steinbusch HW, Verhofstad AA, Emson PC, Spindel E. Immunohistochemical support for three putative transmitters in one neuron: coexistence of 5-hydroxytryptamine, substance P- and thyrotropin releasing hormone-like immunoreactivity in medullary neurons projecting to the spinal cord. Neuroscience 6: 1857–1881, 1981. doi:10.1016/0306-4522(81)90028-2. 6170907

[22] Liu WY, Liu H, Aggarwal J, Huang ZL, Horner RL. Differential activating effects of thyrotropin-releasing hormone and its analog taltirelin on motor output to the tongue musculature in vivo. Sleep 43: zsaa053, 2020. doi:10.1093/sleep/zsaa053. 32227104 PMC7487885

[23] Khomane KS, Meena CL, Jain R, Bansal AK. Novel thyrotropin-releasing hormone analogs: a patent review. Expert Opin Ther Pat 21: 1673–1691, 2011. doi:10.1517/13543776.2011.623127. 22017410

[24] Kinoshita K, Yamamura M, Sugihara J, Suzuki M, Matsuoka Y. Taltirelin hydrate (TA-0910): an orally active thyrotropin-releasing hormone mimetic agent with multiple actions. CNS Drug Rev 4: 25–41, 1998. doi:10.1111/j.1527-3458.1998.tb00039.x.

[25] Messineo L, Carter SG, Taranto-Montemurro L, Chiang A, Vakulin A, Adams RJ, Carberry JC, Eckert DJ. Addition of zolpidem to combination therapy with atomoxetine-oxybutynin increases sleep efficiency and the respiratory arousal threshold in obstructive sleep apnoea: a randomized trial. Respirology 26: 878–886, 2021. doi:10.1111/resp.14110. 34164887

[26] Messineo L, Eckert DJ, Lim R, Chiang A, Azarbarzin A, Carter SG, Carberry JC. Zolpidem increases sleep efficiency and the respiratory arousal threshold without changing sleep apnoea severity and pharyngeal muscle activity. J Physiol 598: 4681–4692, 2020. doi:10.1113/JP280173. 32864734

[27] Smales ET, Edwards BA, Deyoung PN, McSharry DG, Wellman A, Velasquez A, Owens R, Orr JE, Malhotra A. Trazodone effects on obstructive sleep apnea and non-rem arousal threshold. Ann Am Thorac Soc 12: 758–764, 2015. doi:10.1513/AnnalsATS.201408-399OC. 25719754 PMC4418332

[28] Heinzer RC, White DP, Jordan AS, Lo YL, Dover L, Stevenson K, Malhotra A. Trazodone increases arousal threshold in obstructive sleep apnoea. Eur Respir J 31: 1308–1312, 2008. doi:10.1183/09031936.00067607. 18256066 PMC2732198

[29] Veasey SC, Fenik P, Panckeri K, Pack AI, Hendricks JC. The effects of trazodone with l-tryptophan on sleep-disordered breathing in the English bulldog. Am J Respir Crit Care Med 160: 1659–1667, 1999. doi:10.1164/ajrccm.160.5.9812007. 10556137

[30] Eckert DJ, Malhotra A, Wellman A, White DP. Trazodone increases the respiratory arousal threshold in patients with obstructive sleep apnea and a low arousal threshold. Sleep 37: 811–819, 2014. doi:10.5665/sleep.3596. 24899767 PMC4044741

[31] AbdelFattah MR, Jung SW, Greenspan MA, Padilla M, Enciso R. Efficacy of antidepressants in the treatment of obstructive sleep apnea compared to placebo. A systematic review with meta-analyses. Sleep Breath 24: 443–453, 2020. doi:10.1007/s11325-019-01954-9. 31720982

[32] Chen CY, Chen CL, Yu CC. Trazodone improves obstructive sleep apnea after ischemic stroke: a randomized, double-blind, placebo-controlled, crossover pilot study. J Neurol 268: 2951–2960, 2021. doi:10.1007/s00415-021-10480-2. 33625584

[33] Eckert DJ, Younes MK. Arousal from sleep: implications for obstructive sleep apnea pathogenesis and treatment. J Appl Physiol (1985) 116: 302–313, 2014. doi:10.1152/japplphysiol.00649.2013. 23990246

[34] Cummings KJ. Eupnea and gasping in vivo are facilitated by the activation of 5-HT_2A_ receptors. J Neurophysiol 125: 1543–1551, 2021. doi:10.1152/jn.00088.2021. 33760672 PMC8356760

[35] Aton SJ, Seibt J, Dumoulin MC, Coleman T, Shiraishi M, Frank MG. The sedating antidepressant trazodone impairs sleep-dependent cortical plasticity. PLoS One 4: e6078, 2009. doi:10.1371/journal.pone.0006078. 19568418 PMC2699540

[36] Asai H, Asahi T, Yamamura M, Yamauchi-Kohno R, Saito A. Lack of behavioral tolerance by repeated treatment with taltirelin hydrate, a thyrotropin-releasing hormone analog, in rats. Pharmacol Biochem Behav 82: 646–651, 2005. doi:10.1016/j.pbb.2005.11.004. 16368129

[37] Tanabe M, Tokuda Y, Takasu K, Ono K, Honda M, Ono H. The synthetic TRH analogue taltirelin exerts modality-specific antinociceptive effects via distinct descending monoaminergic systems. Br J Pharmacol 150: 403–414, 2007. doi:10.1038/sj.bjp.0707125. 17220907 PMC2189720

[38] Ghanbari R, El Mansari M, Blier P. Electrophysiological impact of trazodone on the dopamine and norepinephrine systems in the rat brain. Eur Neuropsychopharmacol 22: 518–526, 2012. doi:10.1016/j.euroneuro.2011.11.005. 22154666

[39] Ikegami A, Maeda A, Hara H, Yamashita A, Sukamoto T, Ito K. [Effect of trazodone (KB-831) and its metabolites on brain monoamines in rat]. Nihon Yakurigaku Zasshi 93: 145–154, 1989. doi:10.1254/fpj.93.145. 2731805

[40] Rawls WN. Trazodone (Desyrel, Mead-Johnson Pharmaceutical Division). Drug Intell Clin Pharm 16: 7–13, 1982. doi:10.1177/106002808201600102. 7032872

[41] DeVane CL, Boulton DW, Miller LF, Miller RL. Pharmacokinetics of trazodone and its major metabolite m-chlorophenylpiperazine in plasma and brain of rats. Int J Neuropsychopharm 2: 17–23, 1999. doi:10.1017/S1461145799001303. 11281966

[42] Georgotas A, Forsell TL, Mann JJ, Kim M, Gershon S. Trazodone hydrochloride: a wide spectrum antidepressant with a unique pharmacological profile. A review of its neurochemical effects, pharmacology, clinical efficacy, and toxicology. Pharmacotherapy 2: 255–265, 1982. doi:10.1002/j.1875-9114.1982.tb03193.x. 6763207

[43] Dahan A, van der Schrier R, Smith T, Aarts L, van Velzen M, Niesters M. Averting opioid-induced respiratory depression without affecting analgesia. Anesthesiology 128: 1027–1037, 2018. doi:10.1097/ALN.0000000000002184. 29553984

[44] Thirunarayanan N, Nir EA, Raaka BM, Gershengorn MC. Thyrotropin-releasing hormone receptor type 1 (TRH-R1), not TRH-R2, primarily mediates taltirelin actions in the CNS of mice. Neuropsychopharmacology 38: 950–956, 2013. doi:10.1038/npp.2012.256. 23303050 PMC3629383

[45] Bertisch SM, Herzig SJ, Winkelman JW, Buettner C. National use of prescription medications for insomnia: NHANES 1999–2010. Sleep 37: 343–349, 2014. doi:10.5665/sleep.3410. 24497662 PMC3900622

[46] Buysse DJ, Tyagi S. Clinical pharmacology of other drugs used as hypnotics. In: Principles and Practice of Sleep Medicine, edited by Kryger MH, Roth T, Dement WC. St. Louis, MO: Elsevier, Saunders, 2017, p. 432–445.

[47] Algera MH, Cotten JF, van Velzen M, Niesters M, Boon M, Shoham DS, Dandrea KE, van der Schrier R, Dahan A. Respiratory effects of thyrotropin-releasing hormone and its analogue taltirelin on opioid-induced respiratory depression. Br J Anaesth 129: e4–e6, 2022. doi:10.1016/j.bja.2022.03.022. 35459532 PMC9428912

[48] Algera MH, Cotten JF, van Velzen M, Niesters M, Boon M, Shoham DS, Dandrea KE, van der Schrier R, Dahan A. Are thyrotropin-releasing hormone (TRH) and analog taltirelin viable reversal agents of opioid-induced respiratory depression? Pharmacol Res Perspect 10: e00974, 2022. doi:10.1002/prp2.974. 35621218 PMC9137104

[49] Boghosian JD, Luethy A, Cotten JF. Intravenous and intratracheal thyrotropin releasing hormone and its analog taltirelin reverse opioid-induced respiratory depression in isoflurane anesthetized rats. J Pharmacol Exp Ther 366: 105–112, 2018. doi:10.1124/jpet.118.248377. 29674333 PMC5987997

[50] Lelkes Z, Obal F Jr, Alfoldi P, Erdos A, Rubicsek G, Benedek G. Effects of acute and chronic treatment with trazodone, an antidepressant, on the sleep-wake activity in rats. Pharmacol Res 30: 105–115, 1994. doi:10.1016/1043-6618(94)80002-2. 7816739

[51] Paterson LM, Wilson SJ, Nutt DJ, Hutson PH, Ivarsson M. Characterisation of the effects of caffeine on sleep in the rat: a potential model of sleep disruption. J Psychopharmacol 23: 475–486, 2009. doi:10.1177/0269881109104846. 19395429

[52] Park E, Younes M, Liu H, Liu X, Horner RL. Systemic vs. central administration of common hypnotics reveals opposing effects on genioglossus muscle activity in rats. Sleep 31: 355–365, 2008. doi:10.1093/sleep/31.3.355. 18363312 PMC2276746

[53] Younes Y, Park E, Horner RL. Pentobarbital sedation increases genioglossus respiratory activity in sleeping rats. Sleep 30: 478–488, 2007. doi:10.1093/sleep/30.4.478. 17520792

[54] Roda F, Pio J, Bianchi AL, Gestreau C. Effects of anesthetics on hypoglossal nerve discharge and c-Fos expression in brainstem hypoglossal premotor neurons. J Comp Neurol 468: 571–586, 2004. doi:10.1002/cne.10974. 14689487

[55] Shea SA. Behavioural and arousal-related influences on breathing in humans. Exp Physiol 81: 1–26, 1996. doi:10.1113/expphysiol.1996.sp003911. 8869137

[56] Dekin MS, Richerson GB, Getting PA. Thyrotropin-releasing hormone induces rhythmic bursting in neurons of the nucleus tractus solitarius. Science 229: 67–69, 1985. doi:10.1126/science.3925552. 3925552

[57] Fu C, Shi L, Wei Z, Yu H, Hao Y, Tian Y, Liu Y, Zhang Y, Zhang X, Yuan F, Wang S. Activation of Phox2b-expressing neurons in the nucleus tractus solitarii drives breathing in mice. J Neurosci 39: 2837–2846, 2019. doi:10.1523/JNEUROSCI.2048-18.2018. 30626698 PMC6462453

[58] Koivusalo F, Paakkari I, Leppaluoto J, Karppanen H. The effect of centrally administered TRH on blood pressure, heart rate and ventilation in rat. Acta Physiol Scand 106: 83–86, 1979. doi:10.1111/j.1748-1716.1979.tb06373.x. 111471

[59] Thirunarayanan N, Raaka BM, Gershengorn MC. Taltirelin is a superagonist at the human thyrotropin-releasing hormone receptor. Front Endocrinol (Lausanne) 3: 120, 2012. doi:10.3389/fendo.2012.00120. 23087672 PMC3466466

[60] Yamamura M, Kinoshita K, Nakagawa H, Tanaka T, Maeda K, Ishida R. Pharmacological study of TA-0910, a new thyrotropin-releasing hormone (TRH) analog, (I): effects on the central nervous system by oral administration. Jpn J Pharmacol 53: 451–461, 1990. doi:10.1254/jjp.53.451. 2120494

[61] Metcalf G. Regulatory peptides as a source of new drugs—the clinical prospects for analogues of TRH which are resistant to metabolic degradation. Brain Res 257: 389–408, 1982. doi:10.1016/0165-0173(82)90012-1. 6816389

[62] Schuhler S, Warner A, Finney N, Bennett GW, Ebling FJ, Brameld JM. Thyrotrophin-releasing hormone decreases feeding and increases body temperature, activity and oxygen consumption in Siberian hamsters. J Neuroendocrinol 19: 239–249, 2007. doi:10.1111/j.1365-2826.2006.01524.x. 17355315

[63] Hedner J, McCown TJ, Mueller RA, Hedner T, Jonason J, Breese GR. Respiratory stimulant effects by TRH into the mesencephalic region in the rat. Acta Physiol Scand 130: 69–75, 1987. doi:10.1111/j.1748-1716.1987.tb08113.x. 3109212

[64] Nink M, Krause U, Lehnert H, Heuberger W, Huber I, Schulz R, Hommel G, Beyer J. Thyrotropin-releasing hormone has stimulatory effects on ventilation in humans. Acta Physiol Scand 141: 309–318, 1991. doi:10.1111/j.1748-1716.1991.tb09086.x. 1907074

[65] Kinoshita K, Watanabe Y, Asai H, Matsuoka Y. Metabolic abnormalities caused by 3-acetylpyridine in the cerebral motor regions of rats: partial recovery by thyrotropin-releasing hormone. Jpn J Pharmacol 82: 295–300, 2000. doi:10.1254/jjp.82.295. 10875748

